# A simple approach to polymer mixture miscibility

**DOI:** 10.1098/rsta.2009.0215

**Published:** 2010-03-13

**Authors:** Julia S. Higgins, Jane E. G. Lipson, Ronald P. White

**Affiliations:** 1 Department of Chemical Engineering and Chemical Technology, Imperial College, University of London, London SW7 2BY, UK; 2 Department of Chemistry, 6128 Burke Laboratory, Dartmouth College, Hanover, NH 03755, USA

**Keywords:** polymer miscibility, experiments on polymer mixtures, lattice model, prediction of thermodynamic quantities

## Abstract

Polymeric mixtures are important materials, but the control and understanding of mixing behaviour poses problems. The original Flory–Huggins theoretical approach, using a lattice model to compute the statistical thermodynamics, provides the basic understanding of the thermodynamic processes involved but is deficient in describing most real systems, and has little or no predictive capability. We have developed an approach using a lattice integral equation theory, and in this paper we demonstrate that this not only describes well the literature data on polymer mixtures but allows new insights into the behaviour of polymers and their mixtures. The characteristic parameters obtained by fitting the data have been successfully shown to be transferable from one dataset to another, to be able to correctly predict behaviour outside the experimental range of the original data and to allow meaningful comparisons to be made between different polymer mixtures.

## 1. Introduction

Polymeric materials are ubiquitous and irreplaceable not only in the readily recognized banal applications of everyday life but also in high-technology industries such as electronics, aerospace and medicine. The relationship between their molecular structures and their behaviour as materials has been the subject of extensive theoretical and experimental study for many decades. The potential for controlling electrical, mechanical and rheological behaviour not only through choice of chemical repeat unit but through molecular weight, molecular weight distribution, stereo-regularity and degree of branching (and hence crystallinity) has become well understood in recent years through a happy combination of computer modelling, theoretical development and the ability of chemists to synthesize precisely controlled structures to test and validate theory and modelling (for a beautiful example see Heinrich *et al.* (2004)). The understanding of polymer molecules in mixtures—with solvents or with other polymeric species—was early recognized as requiring theoretical developments well beyond the approaches used for mixtures of simple liquids (for a detailed discussion see Koningsveld *et al.* (2001)). Mixtures of polymers are difficult to prepare and control, not only because of their very high viscosity, even at elevated temperatures, but also for thermodynamic reasons. Put simply, the entropy of mixing which so conveniently tends to drive mixing of small molecules becomes a very small contributor for high molecular weight materials, so that all the other parameters contributing to mixing become magnified, and polymers often form thermodynamically stable mixtures only in limited ranges of temperature, pressure and concentration. In practice many polymer mixtures are useful in a state of partial mixing, with heterogeneous structures frozen in by chemical or physical interactions.

**Figure 1.**
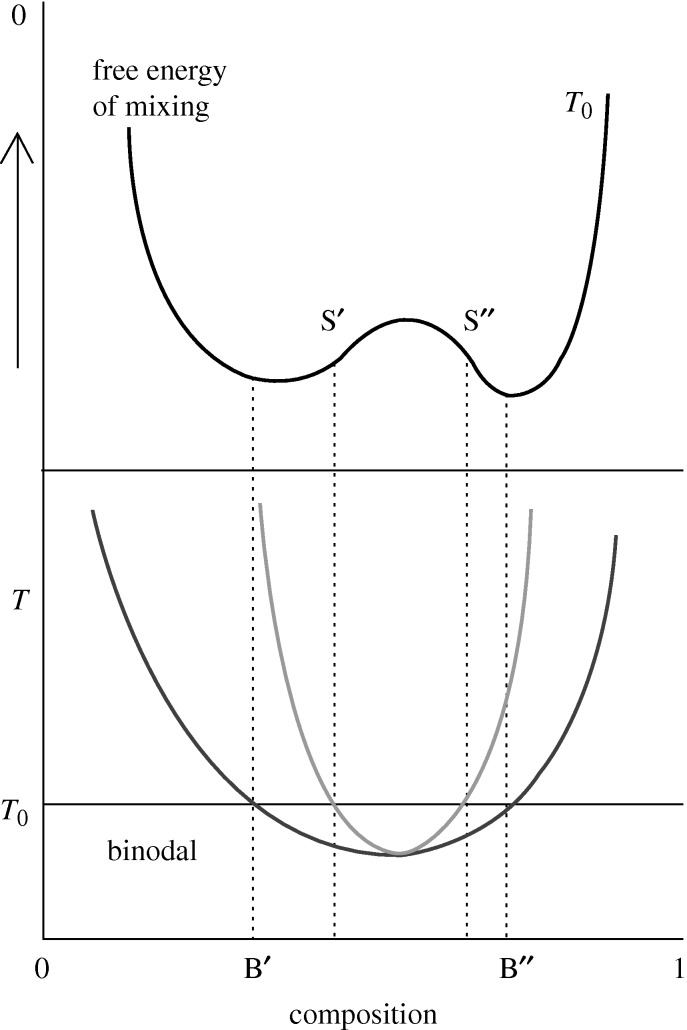
Schematic showing the Gibbs free energy of mixing as a function of composition at a chosen temperature *T*_0_ (upper panel). Given in the lower panel is the corresponding phase diagram showing the binodal (outer, dark grey) and spinodal (inner, light grey) curves; marked are the binodal and spinodal compositions at *T*_0_ (B′, B′′ and S′, S′′) which are related to the features in the free energy curve above.

The phase boundaries and the kinetics of phase separation of polymer blends are very rich areas of investigation, with, additionally, important technological applications. figure 1 shows schematically the variation of the free energy of mixing, *ΔG*_m_, with composition for a typical high molecular weight binary polymer blend and the corresponding phase diagram. Both the binodal (outer, dark grey) and spinodal (inner, light grey) curves are shown in the lower region of figure 1. The binodal denotes the limits of miscibility and is determined by the points of common tangent to the free energy curve, where the chemical potentials of the two co-existing phases will be equal. The spinodal denotes the limits of meta-stability of the system where the curvature changes from positive to negative and the second derivative of *ΔG*_m_ is zero. Inside the spinodal, the system is unstable to all concentration fluctuations and the blend spontaneously separates into co-existing phases via the process known as spinodal decomposition. The process left to develop fully would eventually lead to very large regions of the two co-existing phases; however, the spinodal structure can be frozen in by rapidly cooling the mixture below its glass transition temperature or causing a chemical reaction between the components. In some systems spinodal structures have proved to have interesting material and electrical properties due to the high degree of internal phase boundaries. It is clear that to understand and control such behaviour of polymer mixtures appropriate statistical thermodynamic models are required.

The first, and simplest, statistical thermodynamic model of polymer blends was developed by Flory and Huggins (Flory 1953). The entropy of mixing, *ΔS*_m_, is assumed to be purely combinatorial and is calculated by enumerating the number of arrangements of the molecules on a lattice. The enthalpy *ΔH*_m_ is simply van der Waals energy of contact, and the difference between like and unlike pairs is summarized into a single term, the Flory–Huggins (FH) interaction parameter, *χ*. The free energy of mixing (per monomer), *ΔG*_m_, is then given by1.1
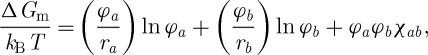

where *r* is the degree of polymerization and *φ* the volume fraction of the species. For a miscible system *ΔG*_m_ must always be negative, and further it must also have positive curvature over the whole concentration range, but for a partially miscible system it will have a region of negative curvature, and hence show the double minima seen in figure 1. It is notable in equation (1.1) that the entropy terms become very small for higher molecular weight polymers because of the terms in *r* in the denominator. Since the enthalpy term is normally positive, owing to unfavourable interactions between unlike pairs of molecules, and the entropy term, which is negative, drives mixing, high molecular weight polymers become less likely to mix. Another feature of equation (1.1) is that it can predict only upper critical solution behaviour (UCST), i.e. phase separation on cooling. The interaction term has an inverse *T* dependence when formulated as in equation (1.1) by FH, and, as a consequence, the enthalpy of mixing becomes less unfavourable at high *T*. In practice, however, most partially miscible blends phase separate on heating, thereby exhibiting a lower critical solution temperature (LCST). This discrepancy between theory and experiment arises mainly because the FH description ignores any volume changes on mixing and simplifies the interactions in the system to a single *enthalpic* parameter. The problems with the FH lattice theory have typically been dealt with arbitrarily in the literature by allowing *χ* to take a complex temperature (and often concentration) dependence. Alternative theories, which take account of volume changes on mixing, have followed a number of different routes—either reformulating lattice models to include holes, for example, or starting with an equation of state formalism which can also account for volume changes. However, all the subsequent modifications to FH theory have not improved the predictive capacity very much, and they all still rely on some sort of fitted interaction parameter, which may contain both enthalpic and entropic terms.

In what follows we present a relatively simple alternative to these approaches and describe some of the successes we have achieved in broadening the range of experimental data which can be used to characterize a system and in being able both to understand and to predict the behaviour of these complex mixtures (Lipson *et al.* 2003; Tambasco *et al.* 2004, 2006; Higgins *et al.* 2005). More generally, recent theoretical treatments of polymer solutions and blends have been developed using a variety of methods, although lattice descriptions tend to dominate. An advantage of lattice theories is that they can be compared with lattice simulations, which, for pragmatic reasons, have dominated the field of polymer simulation results. There have also been recent advances in continuum theories, and it is immediately clear that one gains in complexity and realism at the expense of ease and of potential for use by non-theorists. One feature shared by all approaches is that the microscopic parameters (inherent in any microscopic theory) must be obtained via comparison with (continuum) experimental data.

## 2. A lattice-based equation of state for chain-like molecules

The approach featured in the present work is a lattice-based theory for chain molecule fluids. While still relatively simple, it incorporates several important improvements over FH theory including the effects of compressibility and non-random mixing as well as the general ability to model both the mixture properties and the corresponding properties of the pure components (rather than simply the mixture properties relative to those of the pure components). The theory is based on an integral equation approach wherein segment–segment (pair) distributions are formulated in terms of their corresponding derivatives (with respect to separation distance) and thus are related to the average force between the segments. The solutions for these site–site distributions lead to the calculation of the system’s internal energy, *U*, and integration of *U*(*T*) starting from an athermal reference state (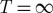) yields a closed-form expression for the Helmholtz free energy and thus, via standard manipulations, the expressions for all of the other thermodynamic properties. As mentioned above, the resulting equation of state is expressed in terms of a set of transferable microscopic parameters, allowing one to *fit* the theory to (a limited set of) known physical properties, and then apply these same parameters in the *prediction* of other properties.

We give here, as an example, the equation of state for the pressure of a pure chain fluid,2.1
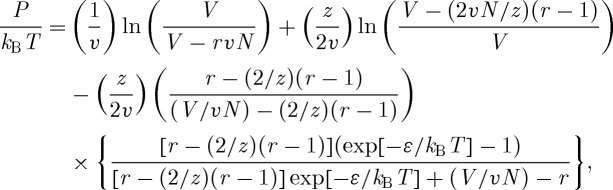

where *V* is the volume, *T* the temperature and *N* the number of chain molecules. Shown in equation (2.1) are the key microscopic parameters; *v* is the volume per lattice site, *r* is the number of lattice sites per chain and *ε* is the site–site nearest neighbour interaction energy. (The lattice coordination number, *z*, is always fixed at a value of 6. Using other values, e.g. *z*=8 or 10, will cause the optimal values of the other parameters to change, but will not appreciably change the overall quality of the fitted, or predicted, properties.) As mentioned above, the present theory incorporates the effects of compressibility; thus, the total volume, *V* , comprises both occupied and empty sites, that is, *V* >*rvN*, where *rv* is the hard core volume occupied by a single chain molecule. The equation of state has a similar form for binary mixtures (i.e. a polymer solution or blend) wherein additional terms are incorporated to account for the presence of a second species. Specifically, three additional parameters are required: *r* and *ε* for the second species, and the parameter *g* which scales the mixed pair interaction energy (*ε*_*ab*_) according to *ε*_*ab*_=*g*(*ε*_*aa*_*ε*_*bb*_)^1/2^. (A single lattice *v* parameter is maintained for the case of a mixture, the value of which is often taken to be a compromise between the optimal *v*’s for the corresponding pure components.) It is important to note that for chain molecules which are long enough (i.e. polymers) the *r* parameter, having been fitted to one molecular weight, can then be easily scaled to predict the properties for systems of different molecular weights. (This is done keeping the values of all the other parameters fixed.) Here, the *r* for any predicted system (‘*r*_predict_’) is scaled proportional to its molecular weight (‘*M*_predict_’) according to *r*_predict_=*M*_predict_(*r*_fit_/*M*_fit_). Examples of the effectiveness of this procedure will be seen in the predicted properties presented in our discussion of the results.

## 3. Results and discussion

A key limitation to the use of any theory, both in analysing experimental data and in making testable predictions, is that a certain amount of data are required in order to characterize the system. The need to specify values for the microscopic parameters of each component (*r*, *ε*, *v*, as discussed in the previous section) is unavoidable, and subsequent predictive ability of the model will be strongly linked to the care with which this process is carried out.

Data on polymer mixtures are available in the literature from many different techniques, but, in terms of direct comparison with equation of state models for mixtures, three types have proved particularly useful. These are pressure–volume–temperature (*PVT*) surfaces for the pure and/or the mixed polymers (e.g. Zoller & Walsh 1995), LCST (or UCST) curves for the mixtures (see many examples in Koningsveld *et al.* (2001)) and zero angle neutron scattering (SANS) data as a function of temperature for the mixtures (see Higgins & Benoit 1994). The zero angle scattered intensity (of any type of radiation) from a mixture *in the one-phase region* is directly proportional to the second derivative with respect to composition of the free energy of mixing. However, for these neutron scattering experiments the hydrogen in one of the components usually has to be replaced by deuterium in order to provide sufficient scattered intensity. We have been exploring and quantifying the non-negligible effect of deuteration of one component in a mixture and will give a brief preview of our results in the discussion that follows. In general the procedure for modelling a blend has been to fit the theory to the pure component *PVT* data in order to obtain the pure component parameters, *ε*, *r* and *v* (e.g. Lipson *et al.* 2003). One single datum from the coexistence curve (i.e. the LCST or the UCST) is then combined with these pure component parameters to obtain the blend parameter, *g*. In Tambasco *et al.* (2004), we explored the advantages of fitting the blend data, either *PVT* or SANS results, directly to give the entire set of pure component parameters as well as *g*, a route which obviates the need for any experimental data on the pure components. The SANS route is particularly appealing, as we have found that even using a small number of data points yields excellent results. However, since the data of choice may not always be available for a system of interest, in the following discussion we explore the use of a variety of kinds of data so as to emphasize the breadth of our approach. It is noted as we now move to a presentation of the results that parameter values and other associated details will be summarized in the captions of the upcoming figures; in cases where they have been made available in other works, we simply cite those sources.

**Figure 2.**
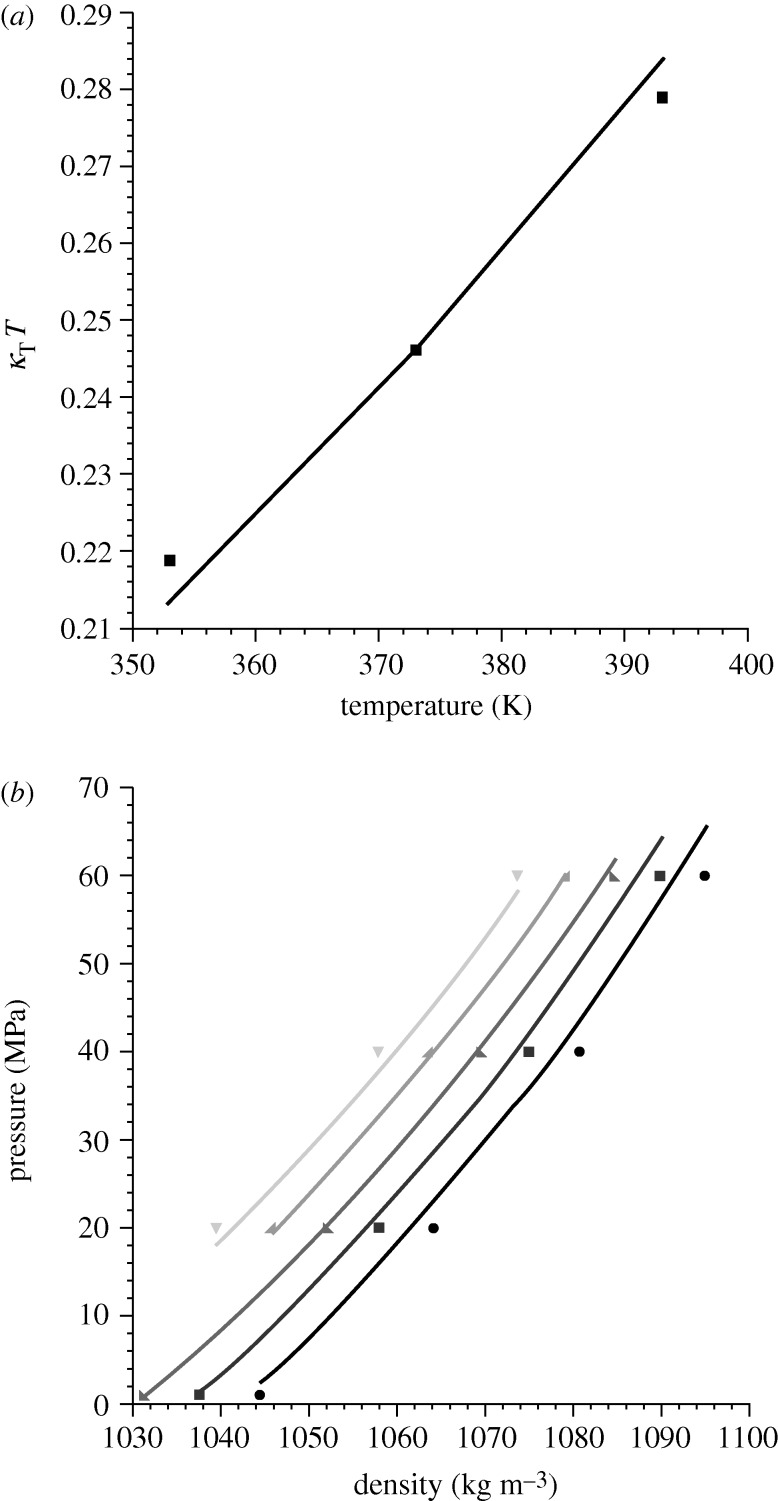
Model fitted to experimental compressibility (*κ*) values (Mark 2007) for (*a*) poly(vinyl acetate) and (*b*) model predictions for the corresponding *PVT* surface; theoretical results are represented by curves and experimental data by symbols. *PVT* data (Zoller & Walsh 1995) are given in the form of pressure–density isotherms corresponding to temperatures 476.8, 485.2, 493.4, 502.1 and 510.2 K. The model parameters (corresponding to a molecular weight of 189 000 g mol^−1^) are given by *r*=15130.4, *v*= 10.170 ml mol^−1^ and *ε*=−2245.5 J mol^−1^.

As mentioned above there are serious limitations to the availability of data, so that one of the first goals in applying the lattice integral equation theory to describe pure polymer fluids was to expand upon the kinds of data which could be used in characterizing individual components. We therefore undertook a series of fits using isothermal compressibilities (*κ*) and thermal expansion coefficients (*α*). These quantities represent derivatives of the *PVT* surface; while they are similar in nature to the complete dataset, fewer experimental data points are required. figure 2*a* shows an example involving poly(vinyl acetate), in which the theory (solid line) was fitted to three experimental points for *κ* in order to determine values for *r*, *v* and *ε*. These parameters were then used to test the lattice theory prediction (solid lines) for the entire *PVT* surface against experimental results (points), as shown in figure 2*b*. The agreement is excellent, providing support for the new characterization route.

**Figure 3.**
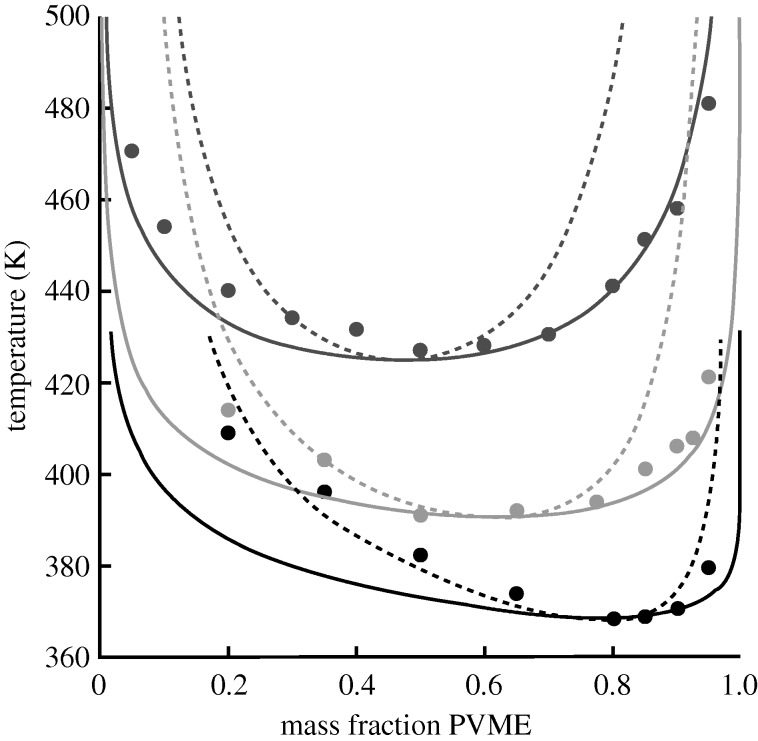
Predicted phase diagrams for the polystyrene/poly(vinyl methyl ether) (PS/PVME) blend at varied molecular weights. The diagrams show both the theoretical binodal (solid) and spinodal (dashed) curves; experimental data are given by points (Nishi & Kwei 1975). The results for three systems are shown, where, from top to bottom, the PS molecular weights (*M*_w_, in g mol^−1^) are 20 400 (dark grey), 51 000 (light grey) and 200 000 (black). In all cases the PVME molecular weight was 51 500. The pure component parameters were obtained from fits to *PVT* data which were weighted for best agreement at low pressure (Zoller & Walsh 1995, PS; and Ougizawa *et al*. 1991, PVME). The parameters for PS are *r*/*M*_w_=0.113767 mol g^−1^ and *ε*=−2144.3 J mol^−1^; those for PVME are *r*/*M*_w_= 0.115343 mol g^−1^ and *ε*=−1948.7 J mol^−1^. A single *v*=7.667 ml mol^−1^ (a compromise, required to model the mixture) applies to both components. Only one point has been fitted (to obtain *g*=1.00121), that being the LCST value for the phase diagram at the bottom (PS *M*_w_=200 000).

Having characterized an experimental system of interest using a minimal dataset is useful only if it allows for predictions regarding behaviour which extend beyond what is already known experimentally. Thus, an important aim of our work has been to exploit the success of the lattice theory in translating our understanding of how a mixture behaves under one set of conditions to new circumstances, going beyond the typical changes in composition or temperature that are usually considered. Examples are shown in the next two figures.

figure 3 shows three sets of experimental coexistence data (symbols) for the polystyrene (PS)/poly(vinyl methyl ether) (PVME) blend, associated with the molecular weights (top to bottom) 20 400/51 500 (dark grey), 51 000/51 500 (light grey), and 200 000/51 500 (black) g mol^−1^. The lattice theory *predictions* are given by the dashed lines for the spinodals and the solid lines for the binodals. Only one datum point has been fit, that being the LCST for the 200 000/51 500 blend in order to determine *g*; the remaining characteristic parameters were obtained from fits to *PVT* data for pure PS and pure PVME. (Note that we use the scaling of the *r* parameter described above to make predictions at varied molecular weights.) As is evident from the figure, the lattice theory does an outstanding job both in predicting the coexistence behaviour for a given blend as well as in predicting the effect of changing molecular weight. The theory is even able of capturing the broadening and shifting of the coexistence curve as the molecular weight of the PS is decreased.

**Figure 4.**
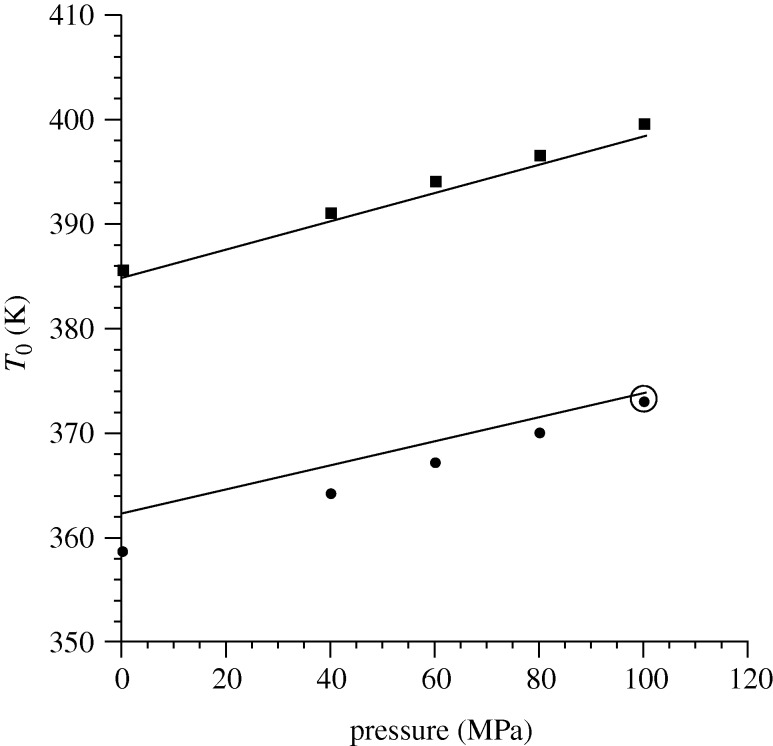
Pressure and molecular weight dependence of the UCST for the polystyrene/ polybutadiene (PS/PB) blend. Model predictions (parameter from Higgins *et al.* (2005, tables 1 and 2)) are given by curves showing the UCST as a function of pressure; experimental results (Tripathi 1979) are given by solid symbols. The upper curve is for a blend with molecular weights of 3900 (PS) and 920 (PB) g mol^−1^. The lower curve is for molecular weights of 1200 (PS) and 2350 (PB) g mol^−1^. One datum point, the experimental point circled on the lower plot at 100 MPa, was used to fit the parameter *g*. Adapted from Higgins *et al*. (2005).

In figure 4, the added complexity of pressure changes is involved, this time for a blend of PS with polybutadiene (PB), which exhibits a UCST for which the pressure dependence has been measured (Tripathi 1979). Once again, the pure component characteristic parameters are obtained by fitting *PVT* data, while *g* derives from a fit to a single datum point (circled in figure 4). One point to note is that the transportability of characteristic parameters for a polymeric melt depends on the molecular weight range of interest (i.e. oligomeric versus polymeric regimes). Parameters determined from data on low molecular weights, say of order 10^3^ g mol^−1^, will not as successfully describe behaviour of melts having molecular weights two or three orders of magnitude greater. Thus, the characteristic parameters for the PS samples associated with figure 4 are different from those used in predicting the results of figure 3. The fact that a single set of parameters (within the oligomeric regime) is able to capture the UCST shift for both blends is therefore significant, even given the apparently small difference in molecular weights. Even more notable is the ability of the theory to predict how miscibility changes over such a large pressure range. While the highest pressure value for one blend UCST was chosen for the *g* determination, similar results are obtained by using another UCST from the combined dataset.

We have demonstrated the success of the lattice integral equation theory in mapping—indeed, predicting—blend miscibility. This presents an opportunity to become more ambitious in terms of what insights to expect from the theory. We have analytic expressions not only for the free energy of mixing, but also for the enthalpy, entropy and volume changes of mixing. Given that such mixing quantities are difficult to obtain experimentally and only infrequently reported, the insights from such predictions are likely to be valuable. It is of course essential to validate such calculations against what data are available, because there is a rich history in statistical thermodynamics of theories which are able to capture free energy effects, yet which over-/underpredict the underlying entropic and enthalpic contributions.

**Figure 5.**
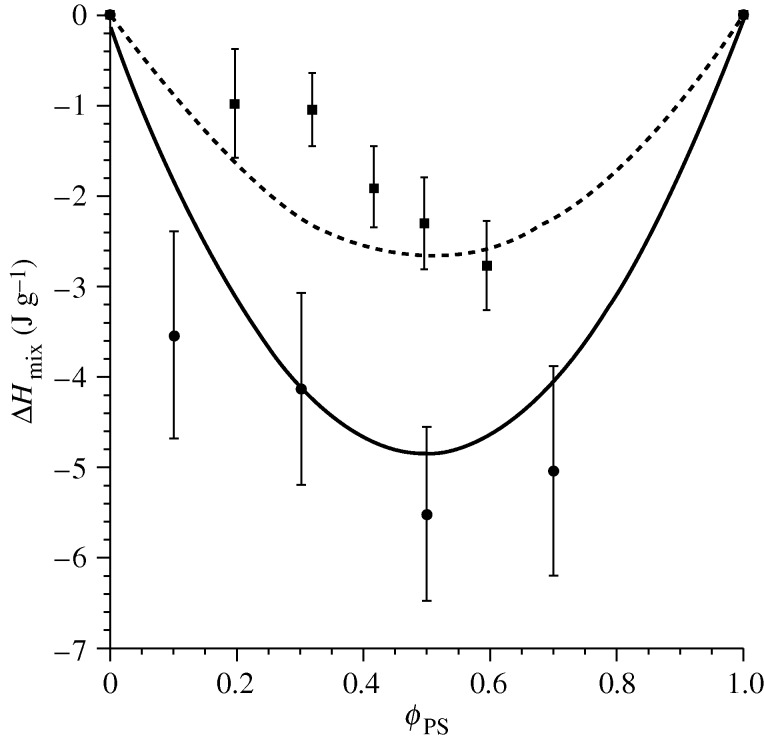
Enthalpy of mixing for the polystyrene/poly(vinyl methyl ether) (PS/PVME) and polystyrene/poly(phenylene oxide) (PS/PPO) blends. For PS/PVME, the model predictions are given by the dashed curve and the corresponding experimental data measured in solution at 50^°^C by squares (Shiomi *et al.* 1990). For PS/PPO, model predictions are given by the solid curve and experimental data measured in solution at 35^°^C by circles (Weeks *et al.* 1977). The model predictions for both systems (PS/PVME and PS/PPO) are derived by fitting to blend *PVT* data (parameters available in Tambasco (2006, tables 4.6 and 4.7)), and, in both cases, datasets for mass fractions of 30/70 and 70/30 were used (Zoller & Walsh 1995).

**Figure 6.**
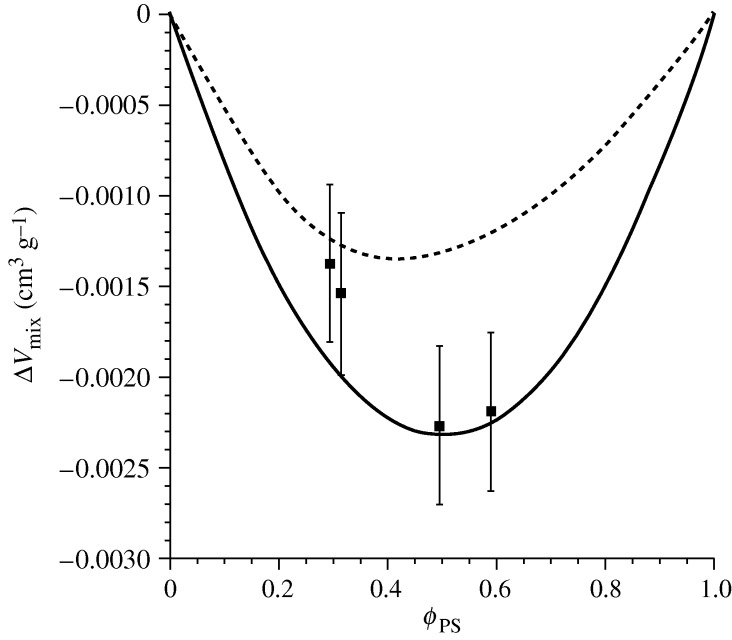
Volume change on mixing for a polystyrene/poly(vinyl methyl ether) (PS/PVME) blend with molecular weights of 110 000 (PS) and 52 800 (PVME) g mol^−1^. Experimental data (symbols) are from Shiomi *et al.* (1990). Two theoretical curves are shown, each representing a different route to the characteristic parameters. The dashed curve is derived from fitting the parameters (Tambasco 2006, table 4.6) to blend *PVT* data (using the datasets for mass fractions of 30/70 and 70/30; Zoller & Walsh (1995)). The solid curve (parameters from Higgins *et al.* (2005, tables 1 and 2)) gives the results obtained by fitting to pure component *PVT* data (Zoller & Walsh 1995) along with a *g*-value obtained by matching to the experimental LCST (Beaucage *et al.* 1991).

In figure 5, we test the ability of the lattice theory to predict enthalpies of mixing for two blends, the LCST mixture PS/PVME and the very miscible blend of PS with poly(phenylene oxide)—PPO. The symbols give experimental data from two separate studies; the error bars give an indication of the difficulty inherent in such experiments and data analysis. The theoretical predictions are shown by the curves; in each case the entire parameter set (including *g*) was determined by fitting to the blend *PVT* surface. The theory not only captures the exothermic nature of mixing in both cases, which is to be expected for such blends, but in fact appears to provide a ‘home’ for the extremely noisy experimental data. This analysis gives confidence that the theory will lead to a deeper understanding of the contributing factors to blend miscibility in systems for which data are scarce.

figure 6 focuses on the volume change on mixing for the well-characterized blend PS/PVME. Once again, such data are difficult to obtain, as demonstrated by both the very small numbers and the very large error bars. Here the theory is put to another sort of test. The two curves represent lattice theory predictions for this blend using two different routes to the characteristic parameters: the dashed line shows the results applying parameters from fitting blend *PVT* data, while the solid line uses results from the fits to pure component *PVT* data along with a *g*-value obtained via a match to the experimental LCST. The difference between the curves indicates the range in prediction resulting from use of different parameter sets. While the discrepancy appears significant note that the volume change on mixing numbers are of the order of 10^−3^ relative to the component volumes, themselves. Interestingly, the two theoretical results appear to provide bounds for the set of experimental values, which neatly fall between the curves.

**Figure 7.**
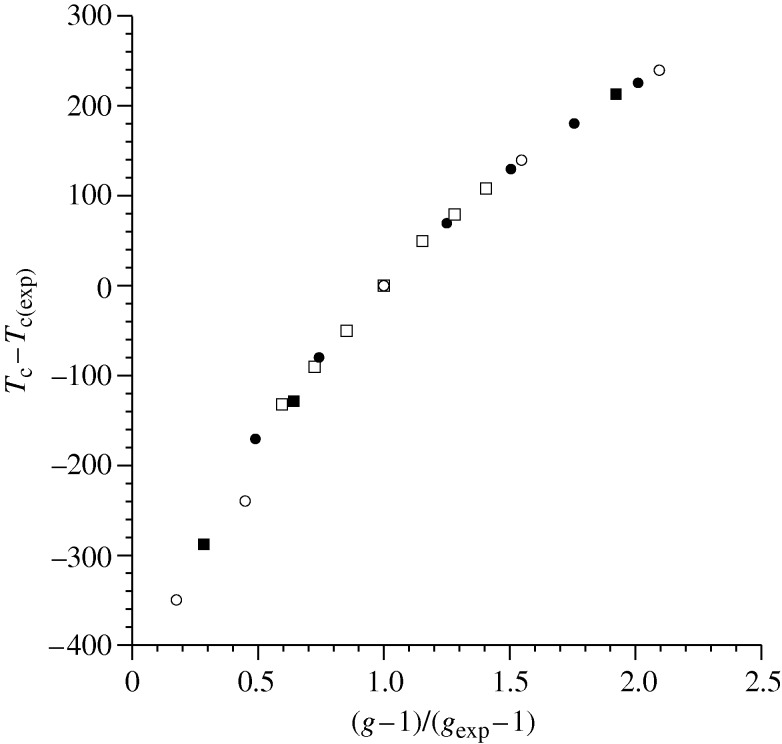
Shifts in the modelled critical temperature as a function of the relative strength of mixed interaction parameter, *g*. *T*_c_ represents the modelled critical temperature while *T*_c(exp)_ is the observed experimental value. *g*_exp_ is the *g*-value that gives *T*_c_=*T*_c(exp)_. Results are shown (parameters from Higgins *et al.* (2005, tables 1 and 2)) for PS/PB (circles *g*−1=−0.0048), polyethylene (PE)/poly(ethlylene–propylene copolymer) (PEP) (squares *g*−1=−0.00078), PS/PVME (diamonds *g*−1=+0.0023), and PS/tetramethylpolycarbonate (TMPC) (triangles *g*−1=+0.00028). The data are adapted from Higgins *et al*. (2005).

Another advantage which should derive from a successful theoretical model of polymer blends is insight which applies across a range of systems. Once having studied a reasonable number of polymeric mixtures, what larger conclusions can be drawn? figure 7 illustrates one attempt to answer such a question, through a plot of the shift in the modelled critical temperature as a function of the relative strength of mixed interaction. The experimental UCST or LCST for each of four polymer blends, indicated using a different symbol, serves as a set of starting points. The UCST blends are PS/PB and polyethylene (PE)/poly(ethlylene–propylene copolymer) (PEP). The LCST blends are PS/PVME and PS/tetramethylpolycarbonate (TMPC). The starting point for each investigation was a fit to pure component *PVT* data to determine all parameters except for *g*, which was obtained in each case by a fit to the experimental critical temperature. Therefore, a common point on the plot for all four systems is *T*_c_−*T*_c(exp)_=0 and (*g*−1)/(*g*_exp_−1)=1. That is, *g*_exp_ is the best-fit value yielding a theoretical critical temperature which matches the experimental value (*T*_c(exp)_) for each system. One point which should be emphasized is that (*g*−1) values for polymer blends are extremely small, being usually of the order of 10^−3^–10^−4^. The very strong sensitivity of the critical temperature to shifts in *g* is typical of descriptions which use this—or a similar—parameter, and has been remarked upon regularly in the literature. Beyond this commonly observed sensitivity, over the range of blends we have studied to date an interesting trend has surfaced: we have invariably found that for LCST blends (*g*−1) values are greater than zero, while for UCST blends (*g*−1) is less than zero. This can be interpreted as the geometric mean underestimating the strength of the mixed interaction for LCST blends, while overestimating it for UCST blends. Therefore, as the caption to figure 7 summarizes, this collection of systems comprises not only both kinds of critical behaviour, but also orders of magnitude differences in (*g*−1) within each of the two subsets.

In order to obtain the results shown in figure 7, for each of the blends the *g* parameter was varied relative to its experimentally fit value and the theory was then used to predict the subsequent shift in the critical temperature. We therefore produce four series of ‘theoretical’ blends, using each of the experimental blends as a starting point. Despite the fact that there are significant differences not only in the interaction strength but also in the nature of the phase separation, the theory predicts the same shift in critical temperature for a given relative shift in mixed interaction. This apparently predictable dependence of miscibility on mixture properties is unexpected and, along with our conclusions linking the sign of (*g*−1) to the type of phase separation, has led us to begin looking more deeply at the underlying theoretical dependence of the free energy and its components on *g*.

As noted earlier, one area of current interest is the effect on melt and blend behaviour upon deuteration of one of the components. For blends, this may be manifest by a significant shift in the critical temperature. The strength and direction of such a shift is currently not well understood; indeed, even the existence of such shifts has not been predicted theoretically. An example of a system that is strongly sensitive to the effects of deuteration is PS/PVME; here, we consider deuteration of the PS component, and thus contrast the two blends, hydrogenated ‘hPS’/PVME and deuterated ‘dPS’/PVME.

**Figure 8.**
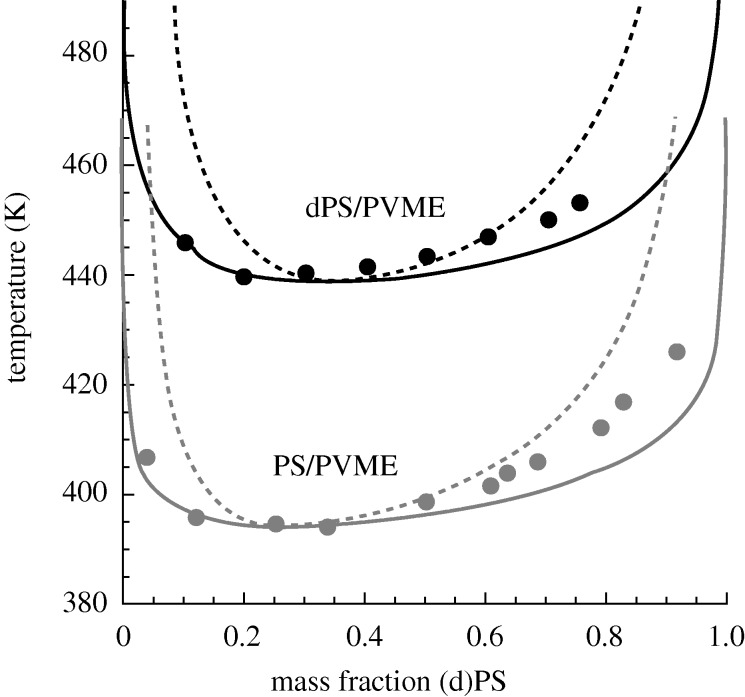
Comparison of hPS/PVME and dPS/PVME phase diagrams. Results are shown for both the theoretical binodal (solid) and spinodal (dashed) curves; experimental data are given by points. The lower diagram (grey) corresponds to the hydrogenated hPS/PVME blend (molecular weights of 120 000/99 000 g mol^−1^; experimental data from Beaucage *et al.* (1991)), the upper diagram (black) corresponds to the deuterated dPS/PVME blend (molecular weights of 119 000/99 000 g mol^−1^; experimental data from Yang *et al.* (1986)). The pure component hPS and PVME parameters are the same as those described in the caption of figure 3. The dPS pure component parameters are *v*=7.667 ml mol^−1^, *r*/*M*_w_= 0.105605 mol g^−1^ and *ε*=−2106.0 J mol^−1^; these were obtained by fitting to SANS data for the dPS/PVME blend (Shibayama *et al*. 1985) where we imposed the same *v* as for hPS and PVME. The *g*-value for the dPS/PVME blend is 1.00098 and was also obtained from the SANS fit. *g* for the hPS/PVME blend is 1.00132 and was obtained by fitting to the experimental LCST in Beaucage *et al*. (1991).

The characteristic parameters for the hPS/PVME blend were obtained by fitting to the pure component *PVT* data (giving *r*, *v* and *ε* for PS and PVME) and then using the experimental LCST value to obtain the mixed interaction parameter, *g*. The phase diagram for hPS/PVME is given in figure 8 (showing both the binodal and spinodal curves) along with the corresponding experimental cloud point data (Beaucage *et al.* 1991). While fitting for *g* pinned the theoretical curve to the experimental results at the critical temperature, the remainder of the phase diagram comprises a true prediction, and it is evident that the theory does a very good job at characterizing the shape of the phase boundary.

The treatment of the dPS/PVME system follows a somewhat different route, although we did use the same characteristic parameters for PVME as were employed above. Noting that *PVT* data on pure deuterated species are essentially unavailable in the literature, we obtained the remaining characteristic parameters by fitting to SANS data (Shibayama *et al.* 1985). Specifically, we employed data for the dPS/PVME blend within the miscible regime and fit to the zero angle scattering intensity (where 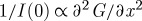) over a range of temperature to obtain the dPS parameters and the value for *g*. The resulting predicted phase diagram for dPS/PVME is shown in figure 8 along with the corresponding cloud point data (Yang *et al.* 1986). The quality of the prediction is excellent as the theory does well in capturing both the location of the LCST and the overall shape of the phase diagram.

Comparing the hPS/PVME and dPS/PVME phase behaviour it is obvious (figure 8) that deuteration of the PS component causes a significant increase in blend miscibility, raising the LCST value by about 40^°^. However, it is important to recognize that, for other blends, deuteration of one of the components does not inevitably lead to increases in miscibility; in some cases, the miscibility decreases, and, in others, little change is observed. Clearly, in the pursuit of explaining these effects, there is room for a predictive theory. Here, given the success in modelling the hPS/PVME and dPS/PVME phase diagrams above, one thing that can be done is to employ the same model parameterization and generate additional properties for inspection. For example, in investigating effects on miscibility it can be illuminating to probe the underlying properties of the corresponding pure components. In the present case this is a particularly valuable potential application for the theory, given the noted lack of pure component experimental data for deuterated species. As noted above, we obtained our parametrization by fitting to SANS data for the dPS/PVME blend, and, thus, we can now ‘back-calculate’ (i.e. predict) the pure dPS properties.

**Figure 9.**
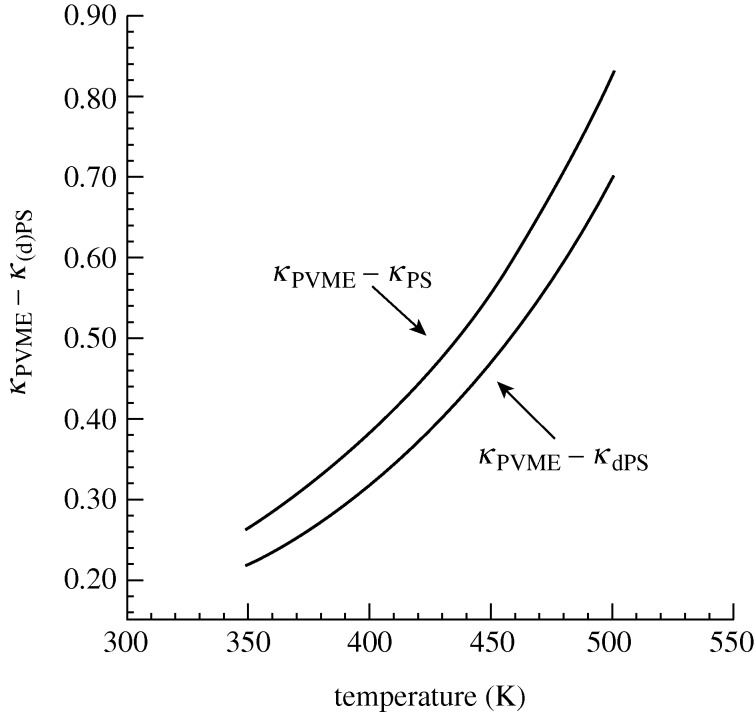
Comparison of the theoretically predicted pure component compressibility (*κ*) values for hydrogenated and deuterated polystyrene (hPS and dPS). Shown are *κ*_PVME_−*κ*_hPS_ (upper curve) and *κ*_PVME_−*κ*_dPS_ (lower curve) as a function of temperature; these are the differences in *κ* of hPS and dPS compared with that of PVME (the other blend component in figure 8). All values are divided by (i.e. are relative to) the value of *κ*_hPS_ at 400 K. All pure component parameters are the same as those in figures 3 and 8. (See those captions for details.)

In rationalizing shifts in LCSTs associated with polymeric systems we note in particular the importance of accounting for compressibility as well as for changes in volume on mixing. This leads naturally to a consideration of properties such as the coefficient of thermal expansion, 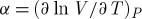, or the compressibility, 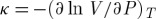. figure 9 shows a comparison of the predicted pure component compressibilities (*κ*) for the hPS/PVME and dPS/PVME systems over the experimentally relevant temperature range. Specifically, we plot the difference in the model *κ* between the two components in each system, that is, *κ*_PVME_−*κ*_hPS_ as compared with *κ*_PVME_−*κ*_dPS_ (both differences are relative to the model value of *κ*_hPS_ at 400 K). The theory predicts that *κ*_PVME_−*κ*_dPS_ is less than *κ*_PVME_−*κ*_hPS_, indicating that the effect of deuterating the PS component causes an increase in its compressibility, thereby bringing its response to changes in pressure closer to that of PVME. This better match in the so-called ‘equation-of-state’ properties is reflected in greater miscibility, here an increase in the LCST.

## 4. Conclusions

In this paper, we have described the application of a simple lattice theory to elucidate the thermodynamic properties of polymeric fluids and mixtures. We have demonstrated that our approach is able to make predictions about thermodynamic quantities not yet measured and experimental conditions not yet studied. In support of these goals, we have illustrated the means by which a minimal amount of experimental data on the pure or mixed components may be used for characterization. We have also shown, through comparison with experiment, that the theory is able to provide insight regarding the thermodynamics of mixing, yielding testable predictions for quantities such as the volume, enthalpy and entropy changes of mixing.

In terms of characterization, we have demonstrated that there is considerable breadth in the types of data which may be used. Thus, *PVT* surfaces and various derivatives of these have been fitted for both single components and binary mixtures. Light scattering data for phase boundaries, critical points of binary mixtures and small angle neutron scattering data from blends in the one-phase region have all been analysed and the characteristic parameters extracted. These have been shown to successfully transfer from one experimental system to another, and to predict correctly behaviour outside the original experimental range. In the course of these studies we have found that the mixing parameter *g* exhibits trends which allow meaningful comparisons of different blends. We anticipate that pursuing this line of work will yield a deeper understanding of the balance of thermodynamic factors which influence mixing.

Our theoretical approach has also allowed us to probe the nature of the shift in phase boundaries caused by deuteration of one component in a blend; we have presented some preliminary results in this paper. We plan to explore further the possible correlation between shifts in miscibility and changes in the compressibilities of the components, with the goal of advising experimentalists regarding the effects of deuteration before they choose to embark on costly neutron scattering studies.

Finally, with a theoretical description in hand that exhibits true predictive ability, the potential to link microscopic structure to macroscopic behaviour of polymer blends is expanded considerably. In particular quantities not easily accessible experimentally can now be computed. We have validated this by comparing calculations with some limited experimental data for Δ*H*_m_ and Δ*V*_m_. We are now poised to explore the impact of changes in local structure and bonding on both the entropy and enthalpy of mixing, so as to gain insight into the nature of the molecular interactions involved as polymeric species mix.
